# Improved longitudinal length accuracy of gross tumor volume delineation with diffusion weighted magnetic resonance imaging for esophageal squamous cell carcinoma

**DOI:** 10.1186/1748-717X-8-169

**Published:** 2013-07-06

**Authors:** Dong-Liang Hou, Gao-Feng Shi, Xian-Shu Gao, Junichi Asaumi, Xue-Ying Li, Hui Liu, Chen Yao, Joe Y Chang

**Affiliations:** 1 Department of Radiation Oncology, Peking University First Hospital, Beijing 100034, China; 2 Department of Radiation Oncology, Beijing Shijitan Hospital, Capital Medical University, Beijing 100038, China; 3 Department of Radiology, Hebei Medical University Fourth Hospital, Shijiazhuang 050011, China; 4 Department of Oral and Maxillofacial Radiology, Field of Tumor Biology, Okayama University Graduate School of Medicine, Dentistry and Pharmaceutical Sciences, 5-1, Shikata-cho, 2-Chome, Okayama 700-8525, Japan; 5 Department of Medical Statistics, Peking University First Hospital, Beijing 100034, China; 6 Department of Radiation Oncology, The University of Texas MD Anderson Cancer Center, Houston, TX 77030, USA

**Keywords:** Esophageal squamous cell carcinoma, Magnetic resonance imaging, Diffusion weighted imaging, Gross tumor volume

## Abstract

**Background:**

To analyze the longitudinal length accuracy of gross tumor volume (GTV) delineation with diffusion weighted magnetic resonance imaging for esophageal squamous cell carcinoma (SCC).

**Methods:**

Forty-two patients from December 2011 to June 2012 with esophageal SCC who underwent radical surgery were analyzed. Routine computed tomography (CT) scan, T2-weighted MRI and diffusion weighted magnetic resonance imaging (DWI) were employed before surgery. Diffusion-sensitive gradient b-values were taken at 400, 600, and 800 s/mm^2^. Gross tumor volumes (GTV) were delineated using CT, T2-weighted MRI and DWI on different b-value images. GTV longitude length measured using the imaging modalities listed above was compared with pathologic lesion length to determine the most accurate imaging modality. CMS Xio radiotherapy planning system was used to fuse DWI scans and CT images to investigate the possibility of delineating GTV on fused images.

**Results:**

The differences between the GTV length according to CT, T2-weighted MRI and pathology were 3.63 ± 12.06 mm and 3.46 ± 11.41 mm, respectively. When the diffusion-sensitive gradient b-value was 400, 600, and 800 s/mm^2^, the differences between the GTV length using DWI and pathology were 0.73 ± 6.09 mm, -0.54 ± 6.03 mm and −1.58 ± 5.71 mm, respectively. DWI scans and CT images were fused accurately using the radiotherapy planning system. GTV margins were depicted clearly on fused images.

**Conclusions:**

DWI displays esophageal SCC lengths most precisely when compared with CT or regular MRI. DWI scans fused with CT images can be used to improve accuracy to delineate GTV in esophageal SCC.

## Background

Accurate delineation of gross target volume (GTV) of esophageal squamous cell carcinoma (SCC) is an important step in radiation therapy. Computed tomography (CT) is the primary modality for delineating GTV and planning radiation treatment. The transverse section margin of esophageal SCC GTV is clear on axial CT scan, while distinguishing the upper and lower margins of the pathologic esophageal wall using CT is difficult, the length of the esophageal SCC is often overestimated when using CT [1]. Although esophagography [2] and endoscopic ultrasonography can determine GTV length more accurately than CT, images obtained using these modalities can’t be fused with CT images in radiotherapy planning systems because no points on the lesion can be matched on the images. Some researchers believe that ^18^ F-fluorodeoxyglucose positron emission tomography (^18^ F-FDG-PET) can more accurately delineate esophageal SCC GTV [3,4]. However, the standardized uptake value (SUV) threshold of the esophageal SCC must be ascertained first when ^18^ F-FDG-PET is employed. As this threshold value is easily influenced by the patient’s physiologic state and scanning condition, and SUV threshold selection criteria vary among different machines and research centers. A previous study [5] concluded that a SUV cut-off of 2.5 may be used to delineate the extent of esophageal SCC GTV, but the conclusion only to use in the research’s hospital because SUV value can not standardized at different hospital or different machine at present.

The pathologic lengths and location of esophageal SCC are important for delineating GTV [6,7]. Therefore, choosing a more accurate imaging modality to determine the location and extent of esophageal SCC GTV is crucial.

Functional magnetic resonance imaging (fMRI) is currently used to obtain information on tumor biology, physiology, and molecular level activity. Diffusion-weighted imaging (DWI), a form of fMRI, can be used to qualitatively and quantitatively analyze the condition of water molecules spread inside the organizations, and it can display microscopic changes such as structural characteristics of pathologic tissues and changes in cell membrane integrity. DWI has long been used to evaluate intracranial lesions [8]. Since DWI exploits the specific water diffusion capacity of biological tissue. The signal intensity depends largely on the presence of barriers to diffusion within the water microenvironment. The signal intensity is estimated to be higher in viable tumor tissue with densely packed diffusion-hindering obstacles than in tissue with less densely packed obstacles, such as tumor necrosis and benign tissue. Based on this theory, one would expect a difference in signal intensity between benign and malignant disease on DW images. Currently, DWI is used to distinguish tumor tissue from non-tumor tissue, assess treatment response and predict treatment outcome. Previous studies have shown that DWI combined with conventional MRI was more accurate for staging ovarian cancer than MRI alone [9,10].

In this prospective study of patients with esophageal SCC who underwent CT, T2-weighted imaging (T2WI) and DWI before radical surgery, we sought to measure the GTV length using these modalities and compare the length with that measured length using pathology.

## Methods

### Patients

The imaging and pathologic data for patients with esophageal SCC who underwent transthoracic esophagectomy with conventional two- or three-field lymphadenectomy performed by the same experienced surgical team from December 2011 to June 2012 were prospectively analyzed. All patients were histopathologically diagnosed with esophageal SCC before surgery. None of the patients received preoperative chemotherapy or radiotherapy. All patients underwent routine pretreatment evaluations, including physical examination, complete blood count, biochemistry surveys of liver and kidney function, electrocardiogram, chest radiography, barium esophagography, esophagogastroscopy with tumor biopsy, ultrasound evaluation of the neck and abdomen, and pulmonary function test. Patients with distant metastasis or diabetes mellitus and those who could not tolerate surgery were excluded. Patients with operable disease underwent CT and MRI in the same position with their arms abducted over their heads. Surgical resection was deemed suitable for patients with esophageal SCC who had no distant metastasis or definite direct tumor invasion to adjacent organs on imaging (T4). Operation was performed within 1–3 days after the CT and MRI scan. All patients gave written informed consent, and the study was approved by the Local Research Ethics Committee (Peking University First Hospital Institutional Ethnic Committee).

### CT scan and delineation of GTV

Patients were asked not to eat or drink for 12 hours before undergoing the plain CT scan, as an empty stomach is generally required. Patients were positioned supine on the scanning bed and were asked to hold their breath in inspiration during the scan. A GE Light Speed Pro32 (GE Healthcare, Milwaukee, Wisconsin, USA) spiral CT scanner was used with a layer thickness of 5 mm, interlayer spacing of 5 mm and field of view (FOV) of 36–38 mm, The scan included the chest entrance of the suprasternal fossa to the lower edge of the liver. All CT images were reviewed by the same experienced radiologist and a radiation oncologist. Thickening of the esophageal wall greater than 5 mm was included in the GTV on CT images. At the axial level, the longitudinal length of the GTV was calculated in terms of the number of CT scanning layers.

### MR scan and delineation of GTV

Before MR scanning, patients were instructed with the breath-holding technique. This technique was practiced to reproduce precisely the same degree of inspiration for each scan series. MR scanning was performed using a 1.5-T clinical scanner (GE Signa 1.5-T Echo Speed Plus with EXCITE II) with a maximum gradient strength of 33 mT/m using an eight-tunnel body phased-array coil. Patients were positioned supine on the scanning bed throughout the examination. Prior to DWI, T2WI were obtained in the transverse plane in each patient. Electrocardiographically gated T2WI fast spin-echo images were obtained using the following parameters: repetition time/echo time, 6R-R interval/72 ms; number of signals acquired, three; matrix, 320 × 224; field of view, 36–38 cm; section thickness, 5 mm; gap, 0.5 mm. DW images were acquired using a single-shot echo-planar imaging sequence with the array spatial sensitivity encoding technique (ASSET) in the transverse plane during breath-holding.

Resolution and signal to noise ratio of DWI images are determined by the b-values. The formula of b values is b = γ^2^G^2^δ^2^(Δ-δ/3), where γ represents gyromagnetic ratio; G presents gradient field strength; δ shows the duration of the gradient field; and Δ represents the time of two gradient interval. Select a different b-value will have an impact on DWI imaging. When the b value is high, the DWI can be more sensitive to water molecules diffusion and can be describe the statues of the water molecule diffusion more accurately. When b value is small, the DWI image resolution is high, but T2-shine through, organ motion, perfusion and other factors can impact more on the DWI images; these are less sensitive to water molecule movement. The b values of normal brain DWI are generally chosen in the range of 800 s/mm^2^ and 1500 s/mm^2^, and the b values of body DWI are usually less than 1000 s/mm^2^. However, at present there are no studies on the application to the esophageal carcinoma. Based on the former research on body DWI and b-values they chose, we obtained DWI images with b values of 0 and 400, 600, 800 s/mm^2^. The components of the applied gradients for diffusion weighting were equal in read, phase, and section orientations to obtain maximum total gradient strength. Other parameters were as follows: repetition time/echo time, 2,450 ms/50.8-67.4 ms; field of view, 44–48 cm; matrix, 128 × 128; section thickness, 5 mm; gap, 0 mm.

All the MRI scans were reviewed by the same experienced radiologist and a radiation oncologist. Thickening of the esophageal wall greater than 5 mm was included in the GTV on T2W images. On DWI scans, high signal intensity regions were included in the GTV. The length of the GTV was calculated according to the number of layers with pathologic changes on T2W images and DWI images.

### Measurement of lesion length using pathologic specimens

The length of the esophagus was measured in situ before cutting the esophagus during surgery. Immediately after the specimen was removed, the esophagus was cut open along the longitudinal axis. So as to not cut though the tumor, the esophagus was stretched to the same length as measured in vivo and pinned on a flat board. Then, the length of the tumor was measured and recorded.

### Image fusion

**Figure 1:**
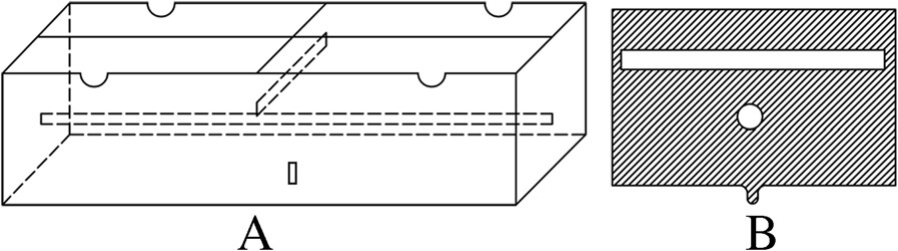
**Diagram of the module. A**: Structural diagram of the CT image and MRI scan amalgamation external controlling point module. **B**: Cross-sectional diagram of the module.

DWI images can not show anatomic structures clearly due to its spatial resolution is limited, while CT images can show anatomic structures clearly because of high spatial resolution. We have to fuse the two images using treatment planning system and delineate GTV on fused images. We chose patients with esophageal SCC who underwent radical radiotherapy to validate the possibility of fusing DWI scans and CT images. We placed three modules [11] (Figure 1A) on the body surfaces of the patients as image match point when they underwent a planning CT scan using GE Light Speed Pro32 spiral CT scanner, and T2WI and DWI scanning using a 1.5-T clinical scanner. The size of the module was 30 mm × 20 mm × 15 mm. The module included one longitudinal pipe and one transverse pipe, which were stereo crossed and mutually perpendicular. The two ends of the longitudinal and transverse pipes were closed and filled with red liquid for double CT and MRI T2WI development. The length of the longitudinal pipe was 30 mm, and the length of the transverse pipe was 20 mm. The inner diameters of the two pipes were 3.5 mm, and the pipe wall thicknesses were 1.5 mm. The vertical projections of the longitudinal pipe and transverse pipe are equipped with relevant longitudinal lines and transverse lines on the surface crossing point, and the longitudinal lines and transverse lines are two crossing lines on the mould surface. The mould can show the internal pipe structure during CT and MRI T2WI scanning. We used the module image as the reference point when fusing DWI and CT scans. During CT scanning, we put the module in the same position on the body surface as during MRI T2WI scanning, making the module longitudinal line parallel with the long axis of the body, and coinciding the transverse line with the CT and MRI T2WI transverse scanning indicatrices. We used the same thickness (5 mm) and FOV (40 cm) for CT and MRI scans. All patients underwent CT and MRI T2WI scanning in the same position and used a respiratory gating device while scanning.

On the module middle cross-section, the transverse pipe on CT images and MRI T2WI scans was the short line (length 20 mm), whereas the longitudinal pipe on images was the small dot (the diameter of the dot was 3.5 mm) (Figure 1B). Modules middle cross-section MRI T2WI scans corresponded to DWI scans on MRI work station as the DWI image fusing base-level image. After collecting imaging data, we transferred the images to the CMS Xio treatment planning system (Version 4.51.02) (Elekta, Stockholm, Sweden) which can reconstruct DWI scans and CT images with volume rendering. We fused base-level DWI scans with base-level CT images manually, while the other DWI scans and CT images were fused automatically. Through such procedures we fused DWI scans with CT images.

### Statistical analysis

Statistical analysis was performed using SPSS software (version 14.0; SPSS, Chicago, Illinois, USA). Paired *t*-tests for normally distributed data were used to assess the differences between groups. Agreement between DWI GTV length and pathologic lesion length was assessed using a Bland-Altman plot. A p value of <0.05 was considered statistically significant.

## Results

We enrolled a total of 42 patients diagnosed with esophageal SCC who underwent CT, T2WI and DWI in the study. Of these 42 patients, 23 were men and 19 were women; their ages ranged from 46 to 77 years (median 58.7 years). Of the cases, 12, 20, and 10 were found in the upper, middle, and lower sections of the chest, respectively. The pathologic types included well-differentiated SCC (n = 18), moderately differentiated SCC (n = 16), and poorly differentiated SCC (n = 8). The CT, T2WI, and DWI scans were complete as were the clinical and pathologic data for all the patients.

We considered pathologic lesion length to be the gold standard for validating the accuracy of tumor delineation via imaging, and we compared the pathologic specimen length with the GTV length determined using CT, T2WI and DWI. The difference between the pathologic lesion length and GTV length on CT-images and T2WI scans was 3.63 ± 12.06 mm and 3.46 ± 11.41 mm, respectively (Table 1). When the diffusion-sensitive gradient b-value was 400, 600, and 800 s/mm^2^, the difference between the pathologic lesion length and the GTV length on the DWI scans was 0.73 ± 6.09 mm, -0.54 ± 6.03 mm and −1.58 ± 5.71 mm, respectively.

**Table 1:** Difference between GTV lengths on scans obtained using various modalities and postoperative pathologic lesion length

**Imaging modality**	**GTV length on images (mm)**	**Difference value between GTV length and pathologic lesion length (mm)**	**p-value of GTV length on images and pathologic lesion length**	**95% confidence interval of difference value**
CT	48.91 ± 18.92	3.63 ± 12.06	0.058	(−20.01-27.27)
T2WI	48.75 ± 20.35	3.46 ± 11.41	0.056	(−18.90-25.82)
b = 400 DWI	46.01 ± 17.21	0.73 ± 6.09	0.444	(−11.21-12.67)
b = 600 DWI	44.75 ± 17.48	−0.54 ± 6.03	0.564	(−12.36-11.28)
b = 800 DWI	43.73 ± 17.27	−1.58 ± 5.71	0.086	(−12.77-9.61)

*Abbreviation*: *CT* Computed tomography, *T2WI* T2-weighted imaging *b* diffusion-sensitive gradient b value.

The difference between the pathologic lesion length and the GTV length using the various modalities was not statistically significant (all *p*>0.05; Table 1).

**Figure 2:**
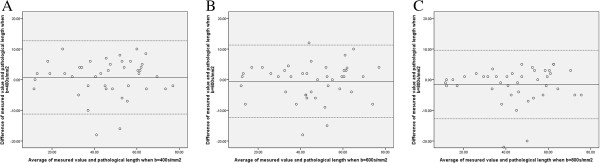
**Bland-Altman plot between the pathologic lesion lengths and the GTV lengths. A**-**C**: Bland-Altman plot displaying the relationship between the pathologic lesion lengths and the GTV lengths measured using DWI scans.

The Bland-Altman plot displays the relationship between the pathologic lesion lengths and the GTV lengths measured using DWI scans. The following figure illustrates the high level of agreement between DWI measurements of esophageal SCC GTV lengths and postoperative pathologic lesion lengths (Figure 2).

**Figure 3:**
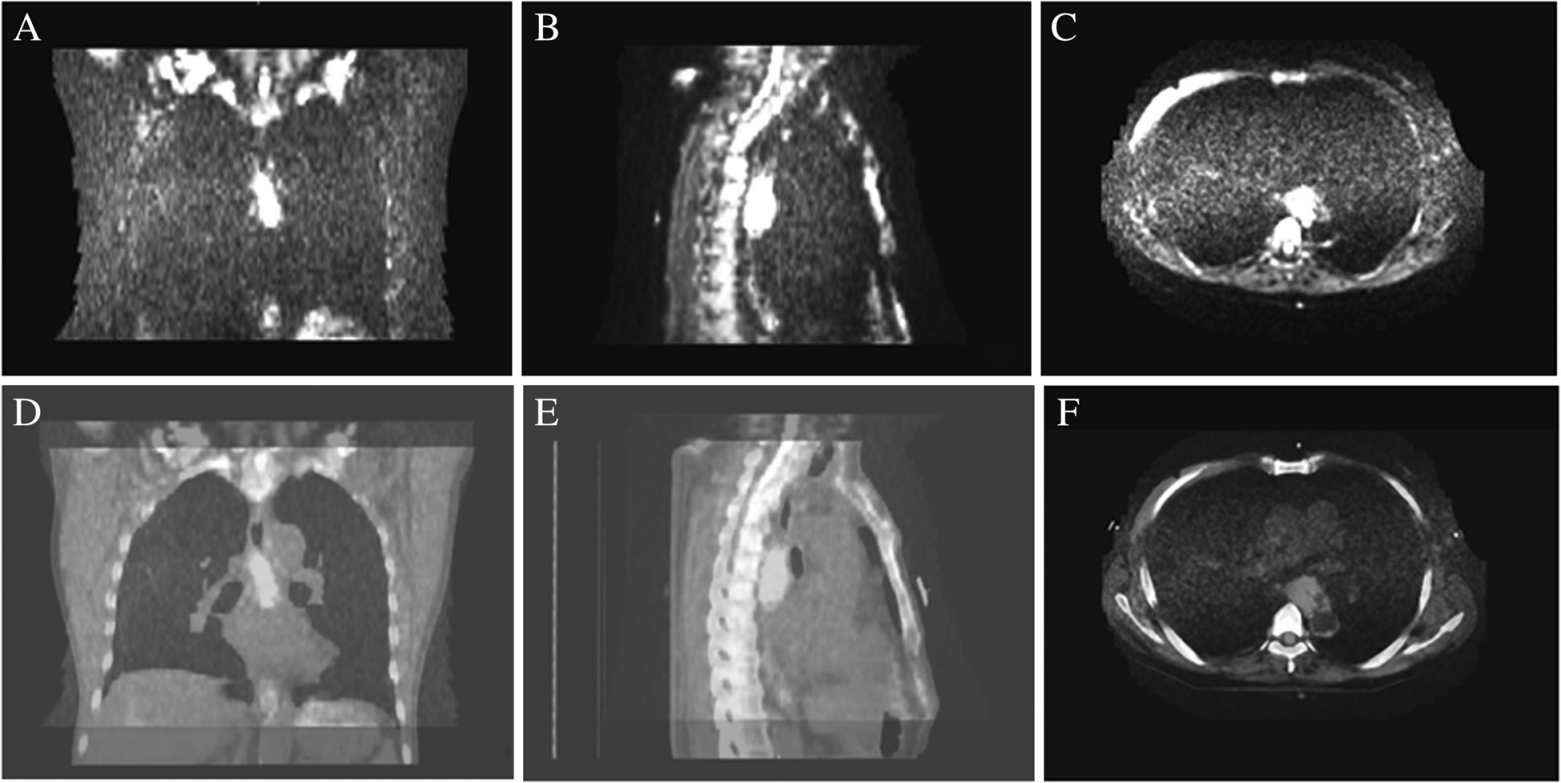
**DWI scans and fused images. A**, **B** and **C** show the coronal, sagittal and transverse images of DWI scans; **D**, **E** and **F** show the coronal, sagittal and transverse images of fused images.

**Figure 4:**
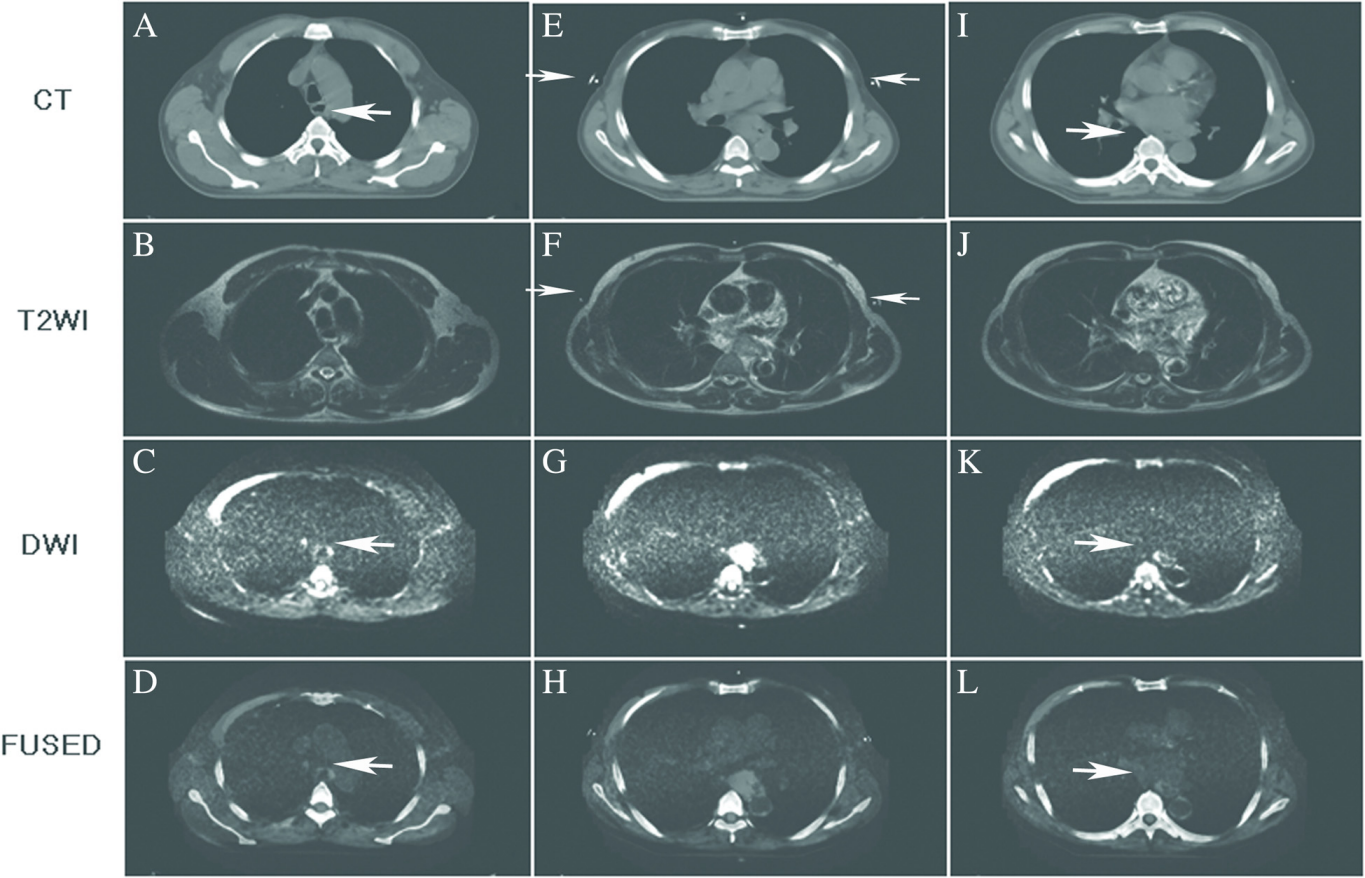
**Different images show the GTV upper boundary, the largest GTV slice and the GTV lower boundary. (A**-**D)** GTV upper boundary (white arrow) on CT images, T2W images, DWI scans and the fused images; In **C** and **D**, the white arrow indicates the upper boundary on DWI scans and fused images. **(E**-**H)** largest GTV slice on images; In **E** and **F**, the white arrow indicates the modules on CT image and T2WI image. **(I**-**L)** GTV lower boundary (white arrow) on CT images, T2W images, DWI scans and fused images; In **K** and **L**, the white arrow indicates the lower boundary on DWI scans and fused images.

We can see from the coronal and sagittal DWI scans and fused images (Figure 3) that esophageal SCC boundary can be depicted clearly on these images, and that DWI scans and CT images can fuse well. On CT images (Figure 4A, I) and T2WI images (Figure 4B, J) the boundary of the esophageal SCC was not clearly apparent, but DWI scans (Figure 4C, K) and fused images (Figure 4D, L) can depict the esophageal SCC GTV upper and lower boundary.

## Discussion

The present study investigated different imaging modalities of delineating esophageal SCC GTV for tumor length determination. Methods tested included CT, T2WI and DWI scans under different diffusion-sensitive gradient b-values. We found that the GTV length measured on DWI scans was close to the pathologic lesion length.

DWI is a non-invasive method for detecting organizational structures at the microscopic level, and mainly detects structural characteristics of the examined organs by measuring the diffusion of water molecules. DWI was first used to diagnose cerebral infarction [12]. Tumors have high cell density and tumor cells have integral membranes that limit the movement of water molecules, thereby contributing to the high signal intensity of lesions on DWI scans. DWI scanning is usually performed at two or more diffusion-sensitive gradient b-values, which indicates the magnitude and duration of the applied gradients and the time between the paired gradients. By varying the amplitudes, lengths and intervals among the diffusion gradients, the sensitivity to the degree of diffusion motion can be altered, and the data can be processed to provide information about actual diffusion distances.

When b-value is high (often>200 s/mm^2^), DWI is more sensitive to water molecule diffusion and can depict the status of water molecule diffusion more accurately than a low b-value. When b-value is low (often<100 s/mm^2^), DWI resolution is high, but T2-shine-through, organ motion, perfusion and other factors can affect the DWI scans and render them less sensitive to water molecule movement than a high b-value. The b-values of normal brain DWI generally range from 800 s/mm^2^ to 1500 s/mm^2^, and the b-values of body DWI are usually less than 1000 s/mm^2^[13,14].

In our practice, we chose different b-values (400, 600, and 800 s/mm^2^) for DWI imaging and delineated GTV on different b-value DWI images. We measured the GTV length and compared it with the postoperative specimen lesion length. Our results showed that GTV on DWI images has good correlation with pathologic lesion length. Likewise, the Bland-Altman plot also illustrated a high level of agreement between DWI measurements of esophageal tumor lengths and pathologic lengths. Of note, although the GTV length measured on different b-value DWI scans correlated with pathologic lesion length, in the majority of the study cases, a b-value of 600 or 800 s/mm^2^ DWI images underestimated the GTV length; this underestimation would lead to inadequate coverage in delineating GTV. As well, when b-value was 400 s/mm^2^, patients needed to hold their breath for about 14 seconds while scanning, whereas when b-value was 600 s/mm^2^ and 800 s/mm^2^, patients needed to hold their breath for about 18 and 20 seconds, respectively. When b-value was 400 s/mm^2^, image resolutions and signal-to-noise ratios were all satisfactory. Given these factors, we recommend choosing a b-value of 400 s/mm^2^ for esophageal SCC DWI scanning; the most suitable b-value for esophageal SCC research, however, still needs to be determined via radiology.

DWI scans must be fused with CT images for radiotherapy planning. We chose patients with esophageal SCC who underwent radical radiotherapy to validate the possibility of fusing DWI scans and CT images. We used imaging fusion software that came with the CMS Xio treatment planning system to register the DWI scans and CT images. The radiotherapy planning system can reconstruct DWI and CT images with volume rendering, and once we fused base-level DWI scans with base-level CT images manually, the other DWI scans and CT images could be fused automatically. Thus, we fused CT images and DWI scans accurately, and delineated GTV on fused images. The limitation of our study is that the DWI images can not show anatomic structures clearly due to its spatial resolution is limit, DWI images have to fuse with CT images with the help of image match point. So the precision of image fusing is important. We used breath-holding technique and hoped through this method, CT and MRI image quality could be less influenced by the movement of the body. Another limitation of this study is lack of lymph nodes assessment through DWI scans because lymph nodes involvement is also important in radiotherapy for esophageal cancer. So the accurate diagnosis of lymph nodes involvement through DWI scans deserves further study.

## Conclusions

In summary, our data suggest that DWI scans can depict esophageal SCC GTV length accurately and also can show GTV upper and lower margins clearly. At the same time, DWI scans can be fused with CT images in radiation treatment planning system, we can use DWI images delineate GTV on fused images.

## Competing interests

None of the authors identify any competing of interests.

## Authors’ contributions

DL Hou and GF Shi contributed equally to this work. DL Hou was the principal investigator of the study and largely wrote the manuscript. GF Shi and HL provided clinical information. XS Gao supervised the research and participated in writing the manuscript, XY Li and CY analysed and interpreted data, JA and JY. Chang reviewed the text of the manuscript. All authors read and approved the final manuscript.
